# Population immunity to pneumococcal serotypes in Kilifi, Kenya, before and 6 years after the introduction of PCV10 with a catch-up campaign: an observational study of cross-sectional serosurveys

**DOI:** 10.1016/S1473-3099(23)00206-2

**Published:** 2023-07-07

**Authors:** Katherine E Gallagher, Ifedayo M O Adetifa, Caroline Mburu, Christian Bottomley, Donald Akech, Angela Karani, Emma Pearce, Yanyun Wang, E Wangeci Kagucia, David Goldblatt, Laura L Hammitt, J Anthony G Scott

**Affiliations:** KEMRI-Wellcome Trust Research Programme, Kilifi, Kenya; Faculty of Epidemiology and Population Health, London School of Hygiene & Tropical Medicine, London, UK; KEMRI-Wellcome Trust Research Programme, Kilifi, Kenya; Faculty of Epidemiology and Population Health, London School of Hygiene & Tropical Medicine, London, UK; KEMRI-Wellcome Trust Research Programme, Kilifi, Kenya; Faculty of Epidemiology and Population Health, London School of Hygiene & Tropical Medicine, London, UK; KEMRI-Wellcome Trust Research Programme, Kilifi, Kenya; KEMRI-Wellcome Trust Research Programme, Kilifi, Kenya; Great Ormond Street Institute of Child Health, University College London, London, UK; Great Ormond Street Institute of Child Health, University College London, London, UK; KEMRI-Wellcome Trust Research Programme, Kilifi, Kenya; Great Ormond Street Institute of Child Health, University College London, London, UK; KEMRI-Wellcome Trust Research Programme, Kilifi, Kenya; Department of International Health, International Vaccine Access Center, Johns Hopkins Bloomberg School of Public Health, Baltimore, MD, USA; KEMRI-Wellcome Trust Research Programme, Kilifi, Kenya; Faculty of Epidemiology and Population Health, London School of Hygiene & Tropical Medicine, London, UK

## Abstract

**Background:**

In Kilifi (Kenya), a pneumococcal conjugate vaccine (PCV10) was introduced in 2011 in infants (aged <1 year, 3 + 0 schedule) with a catch-up campaign in children aged 1–4 years. We aimed to measure the effect of PCV10 on population immunity.

**Methods:**

In this observational study, repeated cross-sectional serosurveys were conducted in independent random samples of 500 children younger than 15 years every 2 years between 2009 and 2017. During these surveys, blood samples were collected by venesection. Concentrations of anti-capsular IgGs against vaccine serotypes (VTs) 1, 4, 5, 6B, 7F, 9V, 14, 18C, 19F, and 23F, and against serotypes 6A and 19A, were assayed by ELISA. We plotted the geometric mean concentrations (GMCs) by birth year to visualise age-specific antibody profiles. In infants, IgG concentrations of 0·35 µg/mL or higher were considered protective.

**Findings:**

Of 3673 volunteers approached, 2152 submitted samples for analysis across the five surveys. Vaccine introduction resulted in an increase in the proportion of young children with protective IgG concentrations, compared with before vaccine introduction (from 0–33% of infants with VT-specific levels over the correlate of protection in 2009, to 60–94% of infants in 2011). However, among those vaccinated in infancy, GMCs of all ten VTs had waned rapidly by the age of 1, but rose again later in childhood. GMCs among children aged 10–14 years were consistently high over time (eg, the range of GMCs across survey rounds were between 0·45 µg/mL and 1·00 µg/mL for VT 23F and between 2·00 µg/mL and 3·11 µg/mL for VT 19F).

**Interpretation:**

PCV10 in a 3 + 0 schedule elicited protective IgG levels during infancy, when disease risk is high. The high antibody levels in children aged 10–14 years might indicate continued exposure to vaccine serotypes due to residual carriage or to memory responses to cross-reactive antigens. Despite rapid waning of IgG after vaccination, disease incidence among young children in this setting remains low, suggesting that lower thresholds of antibody, or other markers of immunity (eg, memory B cells), may be needed to assess population protection among children who have aged past infancy.

**Funding:**

Gavi, the Vaccine Alliance; Wellcome Trust.

## Introduction

Seroepidemiology can be an effective tool to monitor population immunity and guide vaccine policy. When an increase in *Haemophilus influenzae* type b (Hib) cases was observed in England and Wales 7 years after vaccine introduction, the serological pattern of immunity by age suggested that the cause was inadequate boosting of primary anti-Hib responses due to lack of natural exposure. Introduction of a booster dose at age 15 months eliminated the rise in Hib disease incidence.^1^ In France, a similar investigation found that reducing the primary vaccination series from three to two doses had resulted in an immunity gap, which was linked to a rise in cases of invasive Hib disease.^2^

Pneumococcal conjugate vaccines (PCVs) have now been introduced in 164 countries worldwide,^3^ with strong evidence of effectiveness against invasive pneumococcal disease and radiologically confirmed pneumonia.^4^ For *Streptococcus pneumoniae*, both natural colonisation^5,6^ and vaccination elicit serotype-specific serum IgG.^7^ A threshold of serum IgG has been defined as the correlate of protection for invasive pneumococcal disease. In infants this threshold is IgG concentration of 0·35 µg/mL although it may vary by serotype and age.^7,8^ There have only been three population-based pneumococcal serosurveillance studies to date, and only one after vaccine introduction.^9–11^ One study in Malawi showed that in children younger than 5 years, IgG rapidly waned after a primary series of three doses of PCV13.^9^

The effect of PCVs in reducing carriage results in both direct and indirect protection from disease. However, systemic correlates of protection against carriage have been harder to determine, and the mechanism of protection might rely on cellular immunity at the mucosa, secretory IgA and IgG levels, as well as serum IgG levels.^5^ Higher concentrations of IgGs seem to be required for protection against carriage in settings with higher force of infection such as Mali, Nigeria, Nepal, and India compared to places with lower force of infection such as the UK or USA.^12^ This requirement for higher IgG levels might be the reason why vaccine introduction in some settings has reduced disease incidence but has not eliminated carriage of vaccine serotypes (eg, Malawi and Mongolia).^13,14^

In Kenya, a setting with a high force of infection, PCV10 (GlaxoSmithKline, Belgium) was introduced in January, 2011 in a schedule of three doses at 6, 10, and 14 weeks of age, without a booster dose (a 3 + 0 schedule). Any child younger than 12 months who presented to the clinic in 2011 was eligible for three doses of PCV.

Additionally, two catch-up campaigns lasting 1–2 weeks were conducted in Kilifi providing up to two doses of PCV10 to children aged 12–59 months beginning on Jan 31 and March 21, 2011. Within 6 months of introduction, carriage of vaccine serotypes declined by 74% in children younger than 5 years and by 62% in children aged 5–14 years. The decrease in vaccine-type carriage was accompanied by an increase of similar magnitude in non-vaccine serotypes.^15^ The incidence of vaccine-type invasive pneumococcal disease also reduced by 92% in children younger than 5 years, and this incidence has been sustained over time despite a residual vaccine-type carriage of 6% in 2018.^15^

To identify if vaccine introduction without a booster dose led to an immunity gap in older children, we measured the effect of PCV introduction on population immunity over time in children younger than 15 years.

## Methods

### Study setting

This observational study was done in Kilifi county (Kenya), which has a predominantly rural population of 1·4 million (116/km^2^). It was part of the Pneumococcal Conjugate Vaccine Impact Study,^16^ which included about 300 000 residents registered in the Kilifi Health and Demographic Surveillance System (KHDSS).^17^ In 2011 (the year of PCV introduction), vaccine coverage with two or more doses was 80% in children aged 2–11 months, and 66% of children aged 12–59 months had received at least one dose. In 2016, vaccine coverage with two or more doses was 84% in children aged 2–11 months, and 87% of children aged 12–59 months had received at least one dose.^15^

### Data collection and participants

Cross-sectional surveys were conducted before the vaccine was introduced in 2009 and every 2 years thereafter until 2017, always in the cool, dry season. One child per household was selected by independent age-stratified random samples of the KHDSS in 10 age strata (aged 0, 1, 2, 3, 4, 5, 6, 7, 8–9, and 10–14 years) with 50 children in each stratum. Parents or guardians of all participants provided written informed consent and children aged 13–15 years provided written assent. Only those for whom consent was not provided were ineligible. A short questionnaire was administered to collect socio-demographic characteristics and a small (2 mL) blood sample was collected by venesection. The study received ethics approval from the University of Oxford Ethical Review Committee (Number 30–10) and the Kenya Medical Research Institute’s Scientific and Ethical Review Unit (SSC1433).

### Laboratory analysis

Blood samples were separated, aliquoted, and stored at –70°C. Anti-capsular IgG against purified pneumococcal antigens represented in the vaccine (serotypes 1, 4, 5, 6B, 7F, 9V, 14, 18C, 19F, and 23F) and serotypes 6A and 19A was assayed using the WHO reference ELISA, following adsorption with cell wall polysaccharide and 22F polysaccharide at a concentration of 10 mg/mL, as previously described^18^ and detailed online. This assay is based on the original Wyeth assay used to generate the correlate of protection of 0·35 µg/mL. The lower limit of assay quantification was 0·15 µg/mL and IgG concentrations of 0·35 µg/mL or higher were considered protective in infants.^7^

### Statistical analysis

For the primary analysis, to describe population immunity at different ages over time, we tabulated the serotype-specific IgG geometric mean concentrations (GMCs) by survey round and age group (collapsed into four age groups: <1 year, 1–4 years, 5–9 years, and 10–14 years). As samples were collected from ten age strata but the analysis was done in four age groups, we extracted the mid-year population estimates from the KHDSS. These estimates indicated that during the study period, the general paediatric population was evenly distributed by age stratum and that, therefore, no weighting by population size was required to combine estimates into age groups.^17^

We then assessed trends in antibody levels over time. First, we log transformed serotype-specific IgG concentrations and plotted values against age group, by survey round. Second, in order to summarise IgG responses across all ten vaccine serotypes (VTs), we confirmed the log IgG concentrations for the 10 VTs as approximately normally distributed and combined them by creating a Z score, which standardised log(IgG) responses within a common range. We created the Z score using the formula Z=(x – u)/d, where x was the log concentration for the individual, u was the serotype-specific mean log concentration across all timepoints and age groups, and d was the standard deviation of the mean. We combined the 10 Z scores for vaccine serotypes into a single mean Z score for vaccine types, which we plotted against age, by survey round. Graphs were created using the GAMLSS package in R with penalised splines to smooth both the mean and variance.

Within each age group, we analysed changes in antibody levels between survey rounds by conducting a linear regression of serotype-specific log IgG concentrations and of the mean Z score combining responses for all ten VTs. The regression coefficient represented the change in standardised log(IgG) responses across all ten VTs, between survey rounds.

In a secondary exploratory analysis, to examine how IgG changed with age, we used the serial cross-sectional samples to construct three distinct birth cohorts, sampling from each survey the participants who were born within a specific date range, and visualising IgG GMCs over age. We did not take longitudinal samples and so assumed the random sampling from the community was representative at each survey. One birth cohort included children born on or after Feb 1, 2010, and who were therefore younger than 12 months at the time of vaccine introduction and who had a complete record of three doses of PCV. Serotype-specific log IgG responses were visualised using box plots to display the mean and the range of log IgG at each year of age to examine waning of vaccine-induced immunity.

A second birth cohort included children who were born between Feb 1, 2006, and Jan 31, 2010, and who were therefore older than 12 months but younger than 5 years at the time of vaccine introduction and who were vaccinated in the catch-up campaign with one or two doses of PCV. Using a linear regression model, we tested the association between different cohorts (vaccination in infant schedule or in catch up) and standardised mean log(IgG) Z score at 5–9 years of age, controlling for year of age (the age distribution differed between the two birth cohorts even when restricted to children aged 5–9 years).

A third birth cohort included children who were born before Jan 31, 2006, and who were therefore aged 5 years or older at the time of vaccine introduction, and who had never been vaccinated. In this cohort, the standardised mean log(IgG) Z scores for VTs of participants aged 10–14 years at the time of sampling were compared by survey round using linear regression, controlling for year of age, to determine if there was any evidence of a potential immunity gap due to reduced natural exposure. We checked the assumptions of each linear regression model by plotting the residuals to look for non-random patterns. At study inception, 500 individuals, or 50 in each age group, were thought to provide adequate precision around the prevalence estimates (ie, for a proportion of 80%, the precision would be ±4% overall and ±11% in each age stratum).

### Role of the funding source

The funders of the study had no role in study design, data collection, data analysis, data interpretation, or writing of the report.

## Results

Of 3673 volunteers approached, 2438 (66%) consented to the survey and 2152 submitted samples for analysis across the five surveys (appendix p 2). The surveys were similar with respect to age and sex; there were fewer underweight children in 2011 and 2013 than in the other surveys. After vaccine introduction, over 90% of infants were vaccinated appropriately for their age in every survey (table 1).

**Figure 1:**
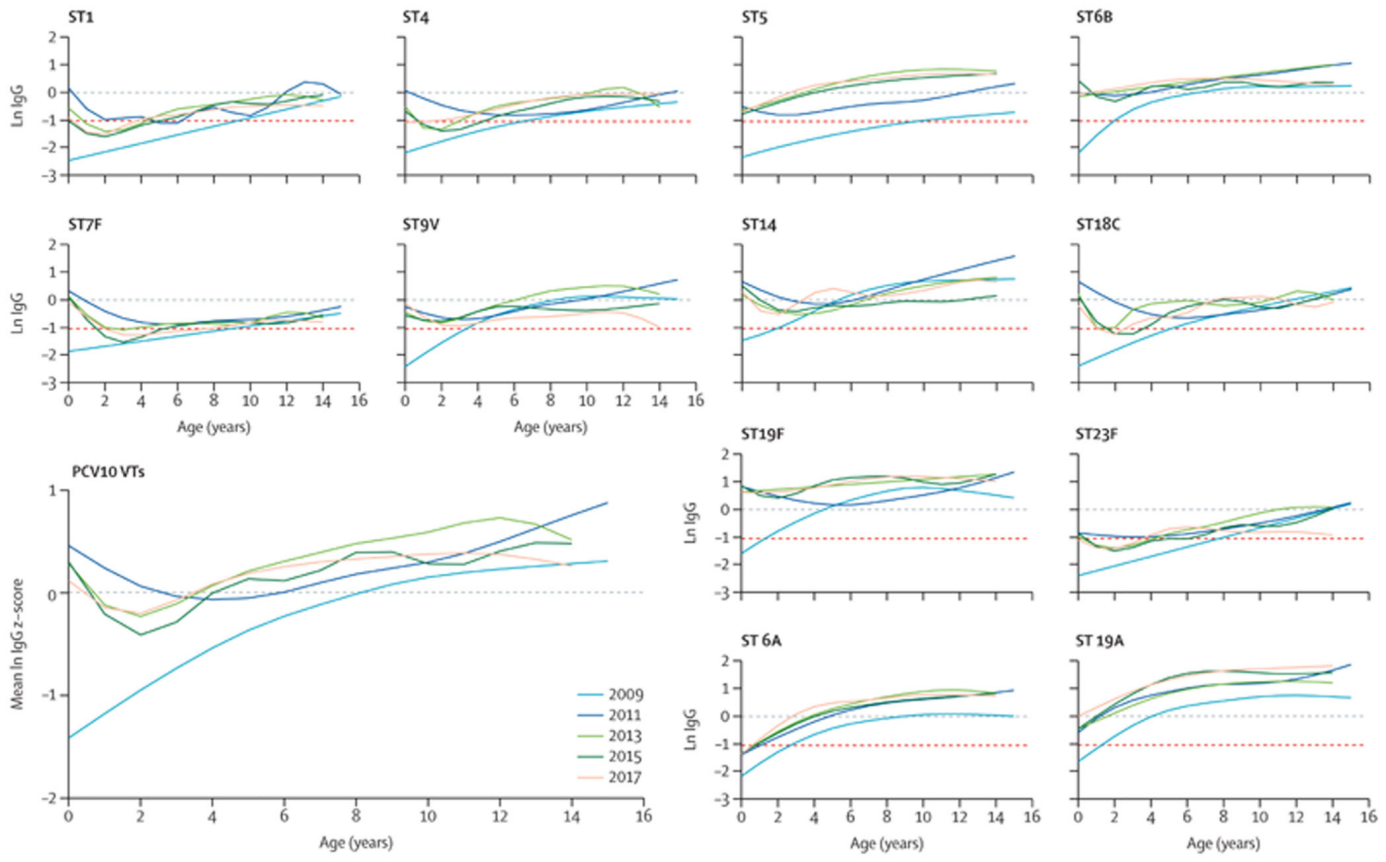
ST-specific log IgG concentrations and standardised z-scores of log IgG concentrations for all PCV10 STs across age, by survey round PCV=pneumococcal conjugate vaccine. ST=serotype. VT=vaccine serotypes. ST-specific IgG concentrations were log transformed (natural log) and converted into a z-score using the formula z=(x – u)/d where x was the raw log concentration, u was the ST-specific mean log concentration across all timepoints and age groups, and d was the standard deviation of the stated mean. The ten z-scores for vaccine STs were then combined into a single mean z-score for vaccine types. Graphs were plotted using the GAMLSS package in R with penalised splines to smooth both the mean and the variance. The red dashed line indicates the correlate of protection in infants (log(0·35 mg/mL)).

In 2009, before vaccine introduction, children accumulated serotype-specific IgGs with age (table 2; figure 1). The proportion of infants (aged <1 year) with protective IgG concentrations against VTs increased from 0–33% across VTs in 2009 (0/36–12/36 children) to 60–94% in 2011 (21/36–34/36 children; appendix pp 3–5), and the GMCs also significantly increased (table 2; appendix pp 6–7). Infants in subsequent survey rounds maintained high IgG GMCs (table 2), and 57–96% of infants (16/28–27/28 children) had protective concentrations against VTs in 2017.

Among children aged 1–4 years targeted with the catch-up campaign in 2011, serotype-specific IgG GMCs and standardised log IgG concentrations for all VTs combined increased from 2009 to 2011 (table 2, figure 1). There is some evidence that by 2017 IgG GMCs among children aged 1–4 years were lower than in 2011 (linear regression coefficient –0·17, 95% CI –0·03 to –0·30; appendix p 7). At all ages, IgG GMCs to non-vaccine serotypes 6A and 19A also increased after vaccine introduction, compared with before vaccine introduction (figure 1).

**Figure 2:**
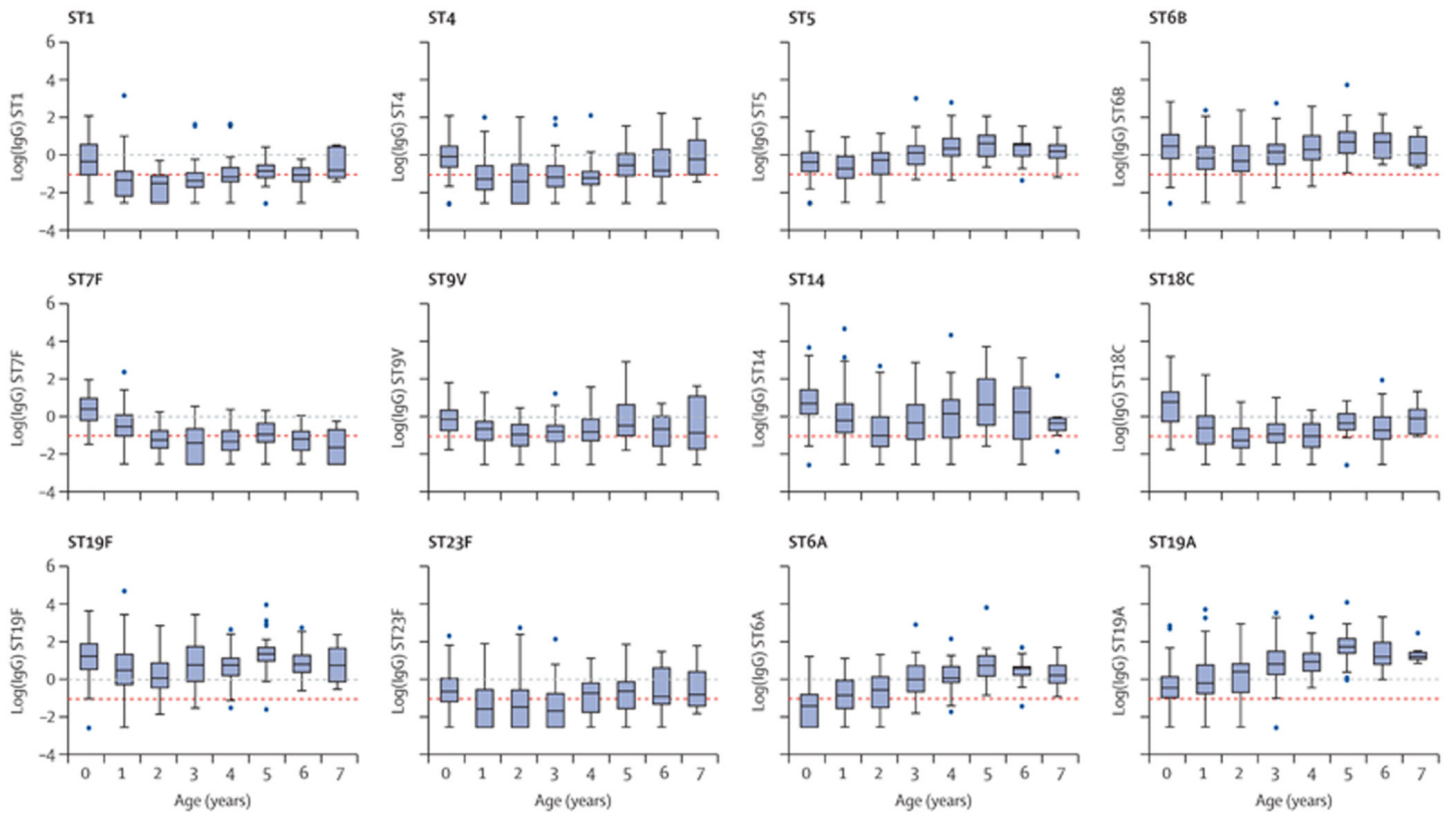
ST-specific log IgG concentrations by age, among children vaccinated in infancy, who received three PCV10 doses PCV=pneumococcal conjugate vaccine. ST=serotype. VT=vaccine serotypes. The log IgG concentrations are displayed in box plots to demonstrate the range in responses. The line across each box represents the median (equivalent to the mean log concentration in a normal distribution) and the outline of the box represents the IQR, where 50% of the data lie. The whiskers represent the range of values, excluding outliers. Outliers are defined as values that lie 1·5 times the IQR above the upper quartile or below the lower quartile. The red dashed line indicates the correlate of protection in infants (log(0·35)).

Among children vaccinated in infancy (ie, born after Feb 1, 2010, and with records of three doses of PCV10), IgG GMCs had waned significantly by the age of 1 year for all ten VTs (figure 2; appendix p 9). IgG concentrations appeared to rise again to the levels achieved in infancy for some serotypes (ie, by age 2 years for serotype 5, by age 4 years for 6B, and by age 5 years for 19F) but for other serotypes, IgG concentrations remained lower than those achieved in infancy until age 6 or 7 years (ie, for 1, 7F, and 18C). For 14 as an example, 93% of vaccinated infants (79 of 85 children) had GMCs higher than 0·35 ug/mL, but this proportion dropped to only 50% (37 of 74 children) of children aged 2 years who had been vaccinated in infancy (appendix pp 8–9).

Despite the presumed reduction in natural exposure to VTs after vaccine introduction, there remained a significant rise in antibodies later in childhood, starting at 2–5 years of age. The standardised mean Z score of IgG concentrations among VTs at age 7 years was not significantly different from post-vaccination concentrations in infancy (appendix p 9).

For non-VT 6A, IgG GMCs increased with age. For non-VT 19A, IgG GMCs were high in infancy and remained high throughout childhood.

**Figure 3:**
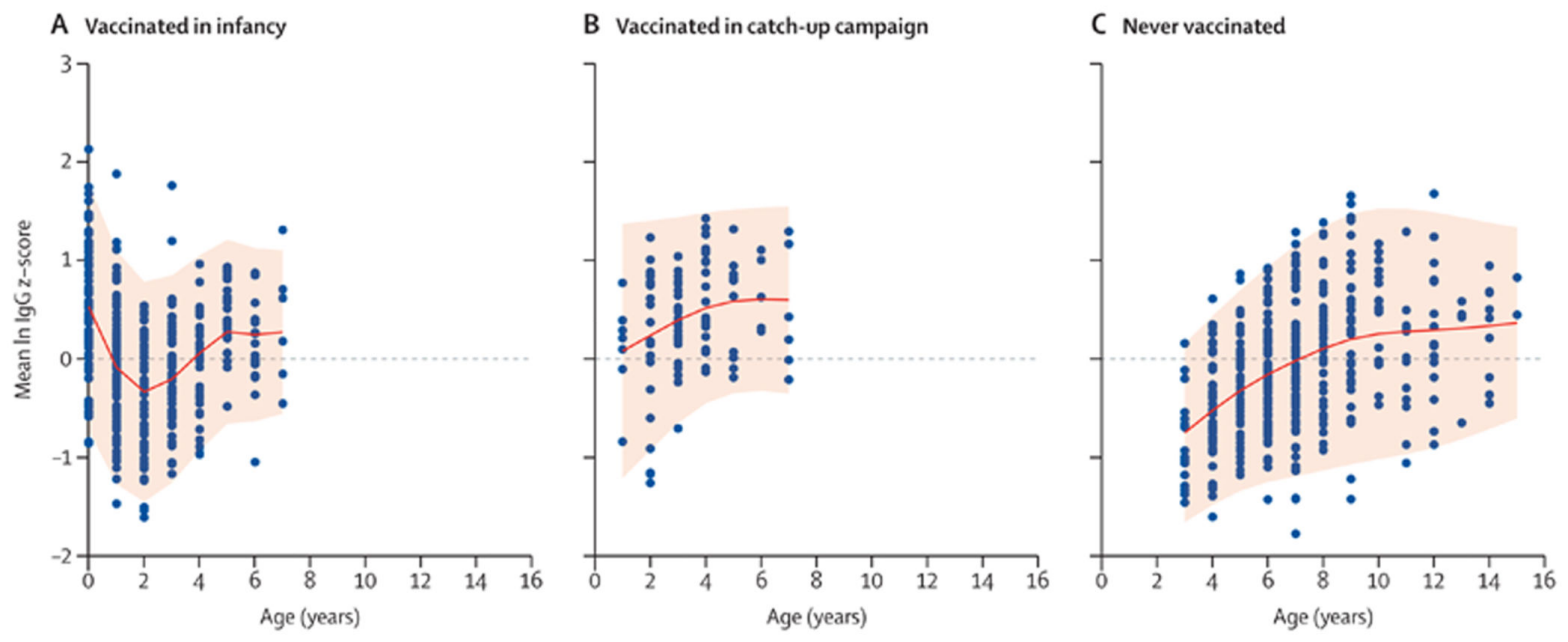
Standardised z-score of IgG concentrations to all vaccine types across age among those vaccinated in infancy (A), those vaccinated in catch-up campaign (B), and those never vaccinated (C) Serotype specific IgG concentrations were log transformed (natural log) and converted into a z-score using the formula z=(x-u)/d where x was the raw log concentration, u was the serotype-specific mean log concentration across all timepoints and age groups, and d was the standard deviation of the stated mean. The ten z-scores for vaccine serotypes were then combined into a single mean z-score for vaccine types. Graphs were plotted using the GAMLSS package in R with penalised splines to smooth both the mean and the variance. Shaded areas indicate the 5th and 95th percentiles.

Children vaccinated in the catch-up campaign did not have significantly different standardised IgG GMC Z scores at age 5–9 years to the Z scores for children vaccinated in infancy with three doses, adjusting for year of age (linear regression coefficient 0·21; 95% CI –0·07 to 0·50). The number of children vaccinated in the catch-up campaign with available data at age 5–9 years was small (n=23), and the corresponding CI was wide (figure 3; appendix pp 10–11).

Among children aged 10–14 years, serotype-specific log IgG concentrations varied by survey round; only 9V showed a potential downward trend over time (table 2; appendix pp 12–13). When all VTs were combined, no change was noted over time in the standardised log IgG Z scores of children in the oldest age group (aged 10–14 years; appendix pp 6–7).

In 2013–17, antibody concentrations in children aged 5–9 years and 10–14 years remained high despite reduced transmission of VTs after vaccine introduction (figure 1). In a sensitivity analysis, among a restricted group of children aged 10–14 years who had records of vaccination and remained unvaccinated, there was also no evidence of a difference in standardised IgG GMC Z scores by survey round, controlling for year of age (appendix pp 13–15).

## Discussion

In the absence of vaccination, serotype-specific antibody to pneumococci in Kenyan children accumulated with age; this has also been documented in the UK and is consistent with age-related exposure to immunising carriage events.^11^ In Kilifi, the introduction of infant PCV10 vaccination in a 3 + 0 schedule, with a catch-up campaign, increased IgG concentrations in infants and young children to that of those aged 5–9 years, protecting this otherwise vulnerable group from invasive pneumococcal disease. Among those vaccinated in infancy, IgG GMCs to seven of the ten vaccine serotypes waned rapidly after vaccination by 1–2 years of age, but they rose again later in childhood. Antibody concentrations remained high in older children, aged 10–14 years, throughout the study. There was no evidence of reduced antibody levels at older ages due to reduced natural exposure over time, despite the rapid decline in VT carriage prevalence after vaccine introduction documented in cross-sectional surveys across all age groups and, presumably, reduced natural boosting in this age group.^15^ Exposure to vaccine serotypes might still accumulate with age due to residual VT carriage and, potentially, sub-dominant carriage,^19^ or B-cell responses to cross-reactive antigens could explain this phenomenon.^20,21^ The data stretch to just 6 years after vaccine introduction. Therefore, a repeated serosurvey at a further timepoint after vaccine introduction might be helpful, as vaccinated children will have aged and represent a larger proportion of the general paediatric population. Unfortunately, we did not perform opsonophagocytic assays and therefore cannot comment on the functionality of the antibody responses we have observed, although opsonophagocytic function has correlated with antibody concentrations in previous studies.^22^

Memory B-cell responses have been shown to play important roles in protection from colonisation and pneumococcal disease. In a human challenge model, circulating capsule-specific IgGs did not correlate with protection against colonisation, whereas levels of specific circulating memory B cells did.^23^ In Malawian children, rates of colonisation correlated with specific memory B-cell responses, and depletion of memory B cells in children with adequate CD4 T-cell counts correlated with increased susceptibility to invasive pneumococcal disease.^20,24^ In Viet Nam, a single dose of PCV10 at 18 months of age stimulated significant rises in circulating B memory cells; however, by 24 months, these levels were only higher than the unvaccinated group for serotype 1 and 18C.^25^

The rapid waning of vaccine-induced IgG has also been reported after PCV13 introduction in Malawi,^9^ which would suggest the results might be generalisable to other similar settings with a 3 + 0 vaccine schedule. Rapid waning of vaccine-induced antibodies in settings with a high force of infection might contribute to the residual VT carriage seen in both Malawi and Kenya, although context-specific contact patterns, vaccination coverage, predominant circulating serotypes, and host susceptibility (eg, malnutrition or HIV) also need to be taken into account when explaining differences in residual carriage across settings.^26^ Despite the rapid waning of IgG after vaccination, previously published data showed that VT carriage declined by 74% (95% CI 65–81) after vaccine introduction, and admissions for invasive pneumococcal disease declined by 92% (78–97) and remained low throughout the study period (3·2 cases per 100 000 person-years) in Kenyan children younger than 5 years.^15^ The population-level mismatch between IgG GMCs and incidence of invasive pneumococcal disease has been documented in a seroprevalence study in the Netherlands at the time of vaccine introduction, where children aged 2–4 years had substantially lower incidence of invasive pneumococcal disease and lower IgG GMCs than children younger than 2 years.^10^ As children’s immune systems mature, they might require less circulating IgG or they might be protected from disease by a combination of indirect protection and rapid memory B-cell responses.^5,15,24^

The IgG concentrations to non-VTs 6A and 19A were higher in the survey rounds conducted after vaccine introduction than in the round before vaccination.

Within 6 months of vaccine introduction in Kilifi, VT carriage had significantly reduced and the prevalence of non-VTs in the nasopharynx increased;^27^ among children younger than 5 years the carriage prevalence of 6A and 19A combined increased from 8·6% in 2009 to 13·2% in 2011.^15^ Although this increase is non-significant (p=0·19), increased natural exposure to non-VTs could be the reason for the increased IgG concentrations to these serotypes in later survey rounds. An alternative hypothesis is that vaccine introduction led to cross-reactive antibodies to these non-vaccine serotypes. However, this hypothesis is not supported by the shape of the antibody profile, which remains a smooth incline with age (without the tick shape indicating vaccine-induced IgG in infants; figure 1). Other non-VTs should be included in future work to compare and contrast responses with the potentially cross-reactive 6A and 19A and assess if the increase in GMCs after vaccine introduction is due to cross-reactivity or increased natural exposure due to serotype replacement.

We combined cross-sectional data into birth cohorts to examine IgG GMCs among children vaccinated in infancy and those vaccinated in a catch-up campaign among children younger than 5 years. We hypothesised that children primed with natural exposure and then vaccinated in the catch-up campaign might have higher IgG later in childhood than those vaccinated in infancy; however, we saw no difference in IgG GMCs among those aged 5–9 years. This finding suggests that a catch-up vaccination might not provide longer-lasting IgGs compared with a vaccination series in infants. Further studies of the impact of catch-up campaigns on carriage and disease are needed to inform strategies in humanitarian settings.

There were some differences in the characteristics of survey participants in each survey round, which might introduce unmeasurable confounding when we compare these cohorts. Approximately 80% of the data on those vaccinated in infancy comes from the final survey round, whereas 20% of the data on those vaccinated during the catch-up campaign comes from the final survey round (appendix p 16). The slightly higher rates of malnutrition in our infant cohort compared with our catch-up cohort will have biased the infants towards lower IgG GMCs.^28^ We have evidence that herd immunity developed rapidly, within 6 months of vaccine introduction,^15,27^ and its effects are therefore likely to be consistent across the survey rounds that occurred after vaccine introduction. None of the participants received infant vaccinations and a boost dose in the catch-up campaign and, therefore, we cannot draw conclusions on the utility of a schedule of primary vaccinations with a boost dose, on the persistence of circulating IgG, nor whether this boost dose would enhance protection from disease. Data from a trial of PCV10 in Viet Nam suggest a 3 + 0 schedule has a non-inferior impact on vaccine-type carriage, to a schedule of 2 primary doses with a booster dose.^29^

Vaccination status was recorded from the electronic vaccine registry^30^ and is likely to be more accurate than relying on immunisation card retention. We analysed the proportion of infants with antibody concentrations above the correlate of protection used for vaccine licensure;^7^ however, we did not analyse the proportion of the population with IgG GMCs above the serotype-specific correlates of protection against invasive pneumococcal disease^8^ nor the correlates of protection against carriage.^12^ These correlates were developed predominantly in high-income settings, and it is uncertain whether they are valid in our setting where residual VT carriage indicates higher concentrations might be necessary for protection. The study was conducted in Kilifi, which was the only county to implement a catch-up vaccination campaign at the time of vaccine introduction. We found similar waning to Malawi,^9^ a setting with a 3 + 0 schedule but no catch-up vaccination; however, due to the catch-up campaign, the results of our study might not be generalisable to the rest of Kenya.

In conclusion, PCV10 vaccination in infancy led to marked increases in VT IgG compared with pre-vaccine periods, at an age when the risk of disease is high. Antibody to most vaccine serotypes waned rapidly in the first 2 years of life; however, in clinical surveillance in Kilifi, vaccine effectiveness against invasive pneumococcal disease was 92% to 4 years of age. This finding suggests these children might be protected by other mechanisms, including lower antibody concentrations or memory B cells and indirect protection. In children 10–14 years of age, antibody concentrations remained high throughout the study period, despite the reduction in natural boosting caused by the decline in transmission of VT pneumococci. Vaccine introduction and reduced exposure to vaccine serotypes have not resulted in reduced population antibody levels at older ages in contrast to what was seen following Hib vaccine introduction to infants in the UK. Continued exposure to vaccine serotypes in residual carriage and potentially as sub-dominant carriage, or memory responses to cross-reactive antigens, could explain this phenomenon. However, the immunity and carriage profile might still be evolving at the population level, and equilibrium following PCV10 introduction with a catch-up vaccination in children younger than 5 years might not become apparent until 7–10 years after introduction.

## Supplementary Material

Supplementary appendix

## Figures and Tables

**Table 1:** Participant characteristics by survey round

	2009 (June 5–Sept 19) (n=469)	2011 (June 25–Nov 4) (n=415)	2013 (June 29–Nov 10) (n=403)	2015 (July 1–Oct 31) (n=437)	2017 (June 28–Nov 8) (n=428)	Total (n=2152)	p value
Age (years)							0 ·99
<1	36 (8%)	36 (9%)	31 (8%)	37 (9%)	28 (7%)	168 (8%)	··
1	42 (9%)	41 (10%)	34 (8%)	44 (10%)	42 (10%)	203 (9%)	··
2	50 (11%)	39 (9%)	36 (9%)	40 (9%)	39 (9%)	204 (10%)	··
3	44 (9%)	40 (10%)	33 (8%)	46 (11%)	50 (12%)	213 (10%)	··
4	53 (11%)	47 (11%)	47 (12%)	44 (10%)	36 (8%)	227 (11%)	··
5	48 (10%)	39 (9%)	43 (11%)	47 (11%)	49 (11%)	226 (11%)	··
6	47 (10%)	44 (11%)	48 (12%)	34 (8%)	48 (11%)	221 (10%)	··
7	48 (10%)	36 (9%)	35 (9%)	48 (11%)	44 (10%)	211 (10%)	··
8–9	49 (10%)	51 (12%)	51 (13%)	49 (11%)	43 (10%)	243 (11%)	··
10–14	52 (11%)	42 (10%)	45 (11%)	48 (11%)	49 (11%)	236 (11%)	··
Sex							0·70
Male	242 (52%)	211 (51%)	200 (50%)	235 (54%)	211 (49%)	1099 (51%)	··
Female	227 (48%)	204 (49%)	203 (50%)	202 (46%)	217 (51%)	1053(49%)	··
MUAC (age **6**–59 months)							<00·0001
<12·5 cm	8/224 (4%)	0	0	4/207 (2%)	10/189 (5%)	22/998 (2%)	··
12·5–13·5cm	21/224 (9%)	1/201 (<1%)	1/177 (1%)	24/207 (12%)	30/189 (16%)	77/998 (8%)	··
>13·5 cm	195/224 (87%)	200/201 (>99%)	176/177 (99%)	179/207 (86%)	149/189 (79%)	899/998 (90%)	··
BMI for age* (age 5–14 years)							<00·0001
<15th percentile	87/244 (36%)	58/212 (27%)	81/222 (36%)	93/226 (41%)	131/233 (56%)	450/1137 (40%)	··
15–85th percentile	141/244 (58%)	140/212 (66%)	120/222 (54%)	118/226 (52%)	73/233 (31%)	592/1137 (52%)	··
>85th percentile	16/244 (7%)	14/212 (7%)	21/222 (9%)	15/226 (7%)	29/233 (12%)	95/1137 (8%)	··
PCV doses for agef† (age <1 year)							<00·0001
Unvaccinated	36/36 (100%)	1/32 (3%)	1/24 (4%)	0	0	38/142 (27%)	··
Partially vaccinated	0	2/32 (6%)	1/24 (4%)	3/32 (9%)	1/18 (6%)	7/142 (5%)	··
Fully vaccinated	0	29/32 (91%)	22/24 (92%)	29/32 (91%)	17/18 (94%)	97/142 (68%)	··

MUAC=mid upper arm circumference. PCV=pneumococcal conjugate vaccine. At the time of the serosurvey participants had their height, weight, and MUAC measured; their vaccination history and sex were recorded. No other characteristics were surveyed. p values were calculated to compare the distribution of data across survey rounds using a χ_^2^_ test. *BMI was calculated for children aged 5–14 years only (inclusive) as weight in kg divided by the square of height (in metres); participants were then classified as to their age-specific and sex-specific percentile using the WHO gender-specific BMI-for-age tables of percentiles. If the BMI lies below the 15th percentile the child is classified as underweight, if BMI is more than the 85th percentile the child is classified as overweight. †Vaccination for age among infants: fully vaccinated was defined as one dose if aged less than 3·5 months, two doses if between 3·5 and 4·5 months, and three doses if >4·5 months; partially vaccinated was defined as just one dose at age 3·5 months or older or just two doses at age 4·5 months or older; unvaccinated was 0 doses under 12 months of age. Missing data indicates children where written records of vaccination were unavailable; this was highest in 2017 (10/28 missing).

**Table 2:** ST-specific IgG GMCs by survey round and age group

	2009	2011	2013	2015	2017	p value*
**ST1**						
<1year	0·08 (0·07–0·10)	1·20 (0·78–1·86)	0·69 (0·45–1·05)	0·41 (0·27–0·62)	0·49 (0·32–0·73)	<0·0001
1–4 years	0·12 (0·11–0·14)	0·43 (0·36–0·51)	0·27 (0·23–0·31)	0·23 (0·20–0·26)	0·26 (0·23–0·31)	<0·0001
5–9 years	0·25 (0·21–0·29)	0·41 (0·34–0·49)	0·56 (0·49–0·64)	0·49 (0·42–0·56)	0·46 (0·41–0·52)	<0·0001
10–14 years	0·50 (0·38–0·67)	0·72 (0·49–1·06)	0·82 (0·61–1·10)	0·69 (0·52–0·92)	0·60 (0·47–0·78)	0·16
**ST4**						
<1 year	0·11 (0·08–0·14)	1·15 (0·80–1·65)	0·80 (0·52–1·23)	0·58 (0·41–0·83)	0·55 (0·37–0·82)	<0·0001
1–4 years	0·20 (0·17–0·23)	0·58 (0·47–0·70)	0·32 (0·27–0·39)	0·27 (0·23–0·32)	0·35 (0·30–0·41)	<0·0001
5–9 years	0·35 (0·29–0·42)	0·45 (0·37–0·55)	0·72 (0·63–0·81)	0·56 (0·47–0·66)	0·71 (0·61–0·83)	<0·0001
10–14 years	0·53 (0·37–0·75)	0·69 (0·50–0·97)	0·99 (0·77–1·26)	0·84 (0·62–1·14)	0·88 (0·68–1·14)	0·028
**ST5**						
<1 year	0·09 (0·08–0·10)	0·81 (0·56–1·17)	0·64 (0·46–0·88)	0·51 (0·39–0·67)	0·53 (0·38–0·75)	<0·0001
1–4 years	0·15 (0·14–0·17)	0·44 (0·39–0·50)	0·81 (0·71–0·92)	0·76 (0·67–0·87)	0·95 (0·83–1·09)	<0·0001
5–9 years	0·26 (0·23–0·30)	0·63 (0·55–0·72)	1·72 (1·53–1·94)	1·34 (1·19–1·50)	1·53 (1·38–1·70)	<0·0001
10–14 years	0·38 (0·29–0·52)	0·95 (0·74–1·23)	2·30 (1·84–2·87)	1·78 (1·45–2·20)	1·95 (1·54–2·47)	<0·0001
**ST6B**						
<1 year	0·10 (0·08–0·12)	1·11 (0·72–1·72)	1·16 (0·75–1·82)	1·65 (1·22–2·23)	1·23 (0·81–1·87)	<0·0001
1–4 years	0·44 (0·37–0·52)	0·88 (0·76–1·02)	1·00 (0·85–1·17)	0·90 (0·77–1·05)	1·13 (0·97–1·32)	<0·0001
5–9 years	0·97 (0·85–1·10)	1·47 (1·30–1·66)	1·56 (1·38–1·77)	1·27 (1·10–1·46)	1·64 (1·44–1·86)	<0·0001
10–14 years	1·23 (0·92–1·65)	2·14 (1·69–2·71)	2·29 (1·79–2·93)	1·33 (1·00–1·77)	1·41 (1·10–1·82)	0·001
**ST7F**						
<1 year	0·15 (0·11–0·20)	1·45 (1·02–2·07)	1·17 (0·73–1·89)	1·12 (0·84–1·50)	0·99 (0·69–1·41)	<0·0001
1–4 years	0·18 (0·16–0·21)	0·59 (0·49–0·72)	0·40 (0·35–0·46)	0·28 (0·24–0·32)	0·33 (0·29–0·38)	<0·0001
5–9 years	0·31 (0·26–0·37)	0·44 (0·37–0·52)	0·40 (0·35–0·47)	0·41 (0·35–0·47)	0·33 (0·29–0·38)	0·007
10–14 years	0·43 (0·32–0·56)	0·52 (0·37–0·72)	0·56 (0·40–0·76)	0·45 (0·34–0·60)	0·46 (0·36–0·59)	0·69
**ST9V**						
<1 year	0·09 (0·08–0·10)	0·91 (0·62–1·33)	0·79 (0·54–1·16)	0·62 (0·49–0·80)	0·92 (0·68–1·25)	<0·0001
1–4 years	0·26 (0·22–0·31)	0·50 (0·42–0·59)	0·51 (0·43–0·59)	0·51 (0·44–0·58)	0·42 (0·36–0·48)	<0·0001
5–9 years	0·78 (0·66–0·92)	0·75 (0·63–0·90)	1·08 (0·95–1·22)	0·78 (0·67–0·90)	0·54 (0·45–0·65)	<0·0001
10–14 years	1·06 (0·83–1·37)	1·34 (0·97–1·86)	1·60 (1·28–2·01)	0·74 (0·57–0·96)	0·60 (0·44–0·81)	<0·0001
**ST14**						
<1 year	0·30 (0·21–0·42)	2·03 (1·36–3·03)	1·57 (0·95–2·59)	1·73 (1·28–2·34)	1·69 (0·99–2·90)	<0·0001
1–4 years	0·38 (0·31–0·47)	1·06 (0·84–1·32)	0·61 (0·50–0·75)	0·74 (0·60–0·91)	0·74 (0·59–0·93)	<0·0001
5–9 years	1·39 (1·14–1·69)	1·09 (0·86–1·38)	0·94 (0·76–1·16)	0·85 (0·68–1·06)	1·30 (1·05–1·61)	0·005
10–14 years	1·78 (1·13–2·79)	2·78 (1·77–4·38)	1·86 (1·25–2·76)	0·93 (0·63–1·40)	1·80 (1·06–3·04)	0·017
**ST18C**						
<1 year	0·09 (0·07–0·11)	2·28 (1·41–3·68)	1·43 (0·83–2·46)	1·30 (0·86–1·96)	0·92 (0·55–1·56)	<0·0001
1–4 years	0·18 (0·16–0·22)	0·84 (0·65–1·09)	0·53 (0·45–0·64)	0·34 (0·29–0·39)	0·38 (0·33–0·44)	<0·0001
5–9 years	0·50 (0·41–0·59)	0·52 (0·44–0·62)	0·91 (0·78–1·06)	0·84 (0·72–0·97)	0·73 (0·64–0·83)	<0·0001
10–14 years	0·94 (0·69–1·28)	0·89 (0·65–1·23)	1·10 (0·84–1·45)	0·90 (0·68–1·20)	0·98 (0·78–1·24)	0·85
**ST19F**						
<1 year	0·18 (0.13·0–25)	2·57 (1·56–4·23)	3·00 (2·03–4·43)	2·91 (2·09–4·06)	2·88 (1·73–4·80)	<0·0001
1–4 years	0·55 (0·47–0·66)	1·57 (1·22–2·02)	1·84 (1·55–2·17)	1·71 (1·43–2·05)	1·84 (1·58–2·15)	<0·0001
5–9 years	1·55 (1·35–1·78)	1·25 (1·02–1·52)	2·74 (2·39–3·13)	3·29 (2·80–3·86)	2·89 (2·51–3·31)	<0·0001
10–14 years	2·00 (1·55–2·58)	2·05 (1·41–2·99)	3·15 (2·41–4·12)	2·64 (1·96–3·56)	3·11 (2·55–3·80)	0·041
**ST23F**						
<1 year	0·10 (0·08–0·13)	0·48 (0·32–0·71)	0·58 (0·35–0·95)	0·49 (0·34–0·69)	0·46 (0·32–0·65)	<0·0001
1–4 years	0·14 (0·12–0·16)	0·38 (0·31–0·45)	0·25 (0·21–0·31)	0·26 (0·22–0·31)	0·27 (0·23–0·33)	<0·0001
5–9 years	0·30 (0·25–0·36)	0·45 (0·39–0·53)	0·54 (0·46–0·65)	0·41 (0·35–0·49)	0·49 (0·41–0·59)	<0·0001
10–14 years	0·64 (0·44–0·94)	0·72 (0·53–0·96)	1·00 (0·71–1·40)	0·65 (0·47–0·89)	0·45 (0·32–0·63)	0·022
**ST6A (non–vaccine ST)**						
<1 year	0·10 (0·08–0·13)	0·21 (0·15–0·30)	0·25 (0·17–0·36)	0·27 (0·19–0·39)	0·23 (0·17–0·32)	<0·0001
1–4 years	0·33 (0·28–0·38)	0·56 (0·49–0·65)	0·72 (0·61–0·85)	0·63 (0·53–0·74)	0·87 (0·74–1·02)	<0·0001
5–9 years	0·80 (0·70–0·91)	1·41 (1·23–1·61)	1·66 (1·48–1·88)	1·50 (1·31–1·71)	1·77 (1·58–1·98)	<0·0001
10–14 years	1·12 (0·85–1·49)	1·97 (1·59–2·45)	2·61 (2·07–3·29)	2·08 (1·62–2·66)	2·17 (1·75–2·69)	<0·0001
**ST19A (non–vaccine ST)**						
<1 year	0·19 (0·13–0·28)	0·42 (0·30–0·59)	0·67 (0·44–1·01)	0·60 (0·44–0·80)	0·97 (0·59–1·58)	<0·0001
1–4 years	0·59 (0·49–0·71)	1·61 (1·39–1·87)	1·31 (1·08–1·58)	1·79 (1·50–2·12)	2·09 (1·76–2·49)	<0·0001
5–9 years	1·45 (1·24–1·69)	2·81 (2·47–3·19)	2·75 (2·42–3·11)	4·87 (4·23–5·61)	4·47 (4·00–4·99)	<0·0001
10–14 years	2·25 (1·68–3·01)	4·00 (3·26–4·91)	3·46 (2·71–4·42)	4·46 (3·54–5·61)	5·73 (4·74–6·92)	<0·0001

Data are GMC in µg/mL (95% CI). ST=serotype. GMC=geometric mean concentration. *p values are derived from linear regression likelihood ratio tests to determine whether there are significant changes in log(IgG) concentrations by survey round, in each age group.

## Data Availability

Data are available from the KWTRP Data Governance Committee (contact via dgc@kemri-wellcome.org) for researchers who meet the criteria for access. Criteria include: the requestor has a disclosed hypothesis and research question that can be answered using the data; and the requestor is affiliated with a reputable research organisation, which has capacity to store and analyse the data according to good clinical practice or good data management practice.

## Research in context

### Evidence before this study

Seroepidemiology has been used to monitor population immunity after the introduction of bacterial conjugate vaccines such as the *Haemophilus influenzae* type b vaccine.

By identifying immunity gaps, findings have influenced vaccine policy through changes to vaccine schedules. Despite widespread introduction of pneumococcal conjugate vaccines into routine immunisation schedules, there has been very little reported serosurveillance after introduction.

We searched for the following terms and their respective subject headings in titles and abstracts on MEDLINE (Ovid SP): (pneumococc* OR Streptococcus Pneumoniae) AND (sero$epi* OR antibod* OR IgG OR immun*) AND (vaccin*) AND (population OR surveillance). Search results were restricted to articles published between Jan 1, 2000, and Nov 20, 2022.

We screened 1465 articles and found three relevant studies. One study reported the distribution of serotype-specific IgG in the UK before vaccine introduction and one study reported the same in the general population of the Netherlands at the time of vaccine introduction. Both documented rising IgG geometric mean concentrations with age. The only study at a timepoint after vaccine introduction was conducted in Malawi in children younger than 5 years. That study suggests rapid waning of IgG directly after a primary series of three doses of pneumococcal conjugate vaccine 13.

### Added value of this study

We report the results of a series of representative cross-sectional surveys that measured the levels of serotype-specific IgG to ten vaccine serotypes and two non-vaccine serotypes among children younger than 15 years in Kilifi, Kenya. The five surveys were conducted before vaccine introduction and every 2 years thereafter up to 6 years after vaccine introduction.

We document rapid waning of vaccine serotype-specific IgGs in children vaccinated in infancy but rising concentrations of these IgG levels later in childhood. We report no evidence of any reduction in concentrations of IgGs to pneumococcal vaccine serotypes in children aged 10–14 years, despite a documented reduction in infection with vaccine serotypes over time.

We report no evidence of vaccine-induced IgGs to non-vaccine types 6A and 19A.

### Implications of all the available evidence

Despite rapid waning of IgG after vaccination, disease incidence in young children in this setting remains low. This low incidence suggests that lower thresholds of antibody, or other markers of immunity (eg, memory B cells), might be needed to assess population protection in children who have aged past infancy. Our study is the first, to our knowledge, to investigate whether reduced natural exposure to vaccine serotypes after vaccine introduction has resulted in a reduction in IgG in older children, aged 10–14 years. We do not find any evidence of such a reduction and, therefore, we can conclude we found no evidence of an immunity gap developing in this group of children. However, continued serosurveillance might be warranted to monitor this protection over time as more of this cohort are exposed to vaccination in infancy. A limitation of our study is the lack of data on antibody function (ie, opsonophagocytosis).
