# Supplementary material for: Population immunity to pneumococcal serotypes in Kilifi, Kenya, before and 6 years after the introduction of PCV10 with a catch-up campaign: an observational study of cross-sectional serosurveys
Source: Lancet Infect Dis. Author manuscript; Available in PMC 2024 Sep 30. (PMC7616650; doi:10.1016/S1473-3099(23)00206-2)
Supplement: Supplementary appendix [file EMS198871-supplement-Supplementary_appendix.pdf]

THE LANCET
Infectious Diseases

Supplementary appendix

This appendix formed part of the original submission and has been peer reviewed. We post it as supplied by the authors.

Supplement to: Gallagher KE, Adetifa IMO, Mburu C, et al. Population immunity to pneumococcal serotypes in Kilifi, Kenya, before and 6 years after the introduction of PCV10 with a catch-up campaign: an observational study of cross-sectional serosurveys. *Lancet Infect Dis* 2023; published online July 7. https://doi.org/10.1016/S1473-3099(23)00206-2.

Population immunity to pneumococcal serotypes in Kilifi, Kenya, before and 6-years after the introduction of PCV10 with a catch-up campaign

Appendix

Supplementary materials

Table of Contents

Supplementary Table 1. Consent rate across the survey rounds 2009-2017....2

Supplementary Table 2. The proportion of children with titres >0.35mg/ml by survey round and age group 3

Supplementary Figure 1. The proportion of participants with IgG GMCs >0.35 ug/ml, across survey rounds, by age group....5

Supplementary Figure 2: Standardised log IgG concentrations (mean z-sores) for all vaccine serotypes combined, by round and age group, with locally weighted regression lines[1]....6

Supplementary Table 3: The change in standardised IgG concentrations for all VTs combined by survey round, estimated using linear regression of mean z-scores....7

Supplementary Table 4. The proportion of children with IgG GMCs >0.35 mg/ml among children vaccinated in infancy (born on or after 1st Feb 2010 and therefore aged<12 months at the time of vaccine introduction on 31st Jan 2011 & who received 3 PCV doses; n=425) 8

Supplementary Table 5. Change in ST-specific log (IgG concentrations) and the mean z-score for all VTs, by age, among those vaccinated in infancy....9

Supplementary Figure 3. Standardised IgG concentrations (z-scores) for all VTs among 5-9 yr olds who a) eligible for infant vaccination and received 3 doses in infancy b) eligible for catch up and received 1 or 2 doses in catch up or c) were ineligible for vaccination and received no vaccination 10

Supplementary Table 6: The association between standardised IgG concentrations (z-scores) for all VTs and cohort of vaccination, among 5-9 year olds 11

Supplementary Figure 4. Log IgG concentrations among all participants age 10-14 over time[1]. [1]Data for this is in table 2 in the main manuscript 12

Supplementary Table 7. ST-specific IgG GMCs among participants aged 10-14 who remained unvaccinated, over time 13

Supplementary Figure 5. Standardised mean z-scores for all VTs among: left) all participants aged 10-14, and; right) those who remained unvaccinated, over time 14

Supplementary Table 8. Linear regression coefficients. Mean standardised z-score of VT GMCs among children 10-14 years of age who were ineligible for vaccination and unvaccinated, by round....15

Supplementary Figure 6: Graphical representation of the number sampled per survey round and contributions to the birth cohort analysis. 16

Supplementary Table 1. Consent rate across the survey rounds 2009-2017

Survey Round	Total Approached	Consented	Refused	Not Available	*% available*	*% consent among those available*	Sample collected	No sample	Lab results	No results
2009	964	605	273	86	*91%*	*69%*	474	131	469	5
2011	826	459	246	121	*85%*	*65%*	421	38	415	6
2013	950	455	305	190	*80%*	*60%*	410	45	403	7
2015	768	475	171	122	*84%*	*74%*	445	30	437	8
2017	796	444	240	112	*86%*	*65%*	436	8	428	8
Total	4304	2438	1235	631	*85%*	*66%*	2186	252	2152	34

Supplementary Table 2. The proportion of children with titres >0.35mg/ml by survey round and age group

Age (years)	2009 n >0.35 ug/ml	% of age group	2011 n >0.35 ug/ml	% of age group	2013 n >0.35 ug/ml	% of age group	2015 n >0.35 ug/ml	% of age group	2017 n >0.35 ug/ml	% of age group
**Vaccine-types**										
**ST1**										
<1	1	**2.8**	32	**88.9**	21	**67.7**	18	**51.4**	19	**67.9**
1-4	17	**9.1**	94	**57.0**	53	**35.8**	46	**28.2**	50	**32.5**
5-9	55	**28.7**	90	**53.3**	122	**69.3**	96	**59.6**	113	**63.5**
10-14	33	**63.5**	30	**71.4**	38	**84.4**	37	**80.4**	37	**75.5**
**ST4**										
<1	4	**11.1**	32	**88.9**	24	**77.4**	27	**75.0**	20	**71.4**
1-4	52	**27.8**	109	**66.5**	64	**42.7**	70	**40.5**	76	**45.5**
5-9	87	**45.3**	97	**57.7**	141	**80.1**	110	**62.9**	138	**75.0**
10-14	33	**63.5**	31	**73.8**	41	**93.2**	38	**79.2**	42	**85.7**
**ST5**										
<1	1	**2.9**	29	**80.6**	22	**71.0**	23	**67.7**	20	**71.4**
1-4	37	**19.7**	106	**63.5**	130	**86.7**	135	**79.0**	143	**87.7**
5-9	79	**41.2**	126	**74.6**	171	**97.2**	171	**97.2**	178	**96.7**
10-14	29	**55.8**	36	**85.7**	45	**100.0**	48	**100.0**	48	**98.0**
**ST6B**										
<1	0	**0.0**	31	**86.1**	27	**87.1**	35	**94.6**	25	**89.3**
1-4	114	**62.0**	141	**84.9**	131	**87.3**	141	**81.0**	147	**88.6**
5-9	167	**87.4**	163	**97.6**	169	**96.6**	163	**93.1**	178	**96.7**
10-14	44	**86.3**	42	**100.0**	44	**97.8**	44	**91.7**	47	**95.9**
**ST7F**										
<1	7	**19.4**	31	**86.1**	25	**80.7**	33	**89.2**	25	**89.3**
1-4	52	**27.8**	115	**69.3**	91	**60.7**	71	**41.3**	75	**45.2**
5-9	84	**43.8**	102	**60.7**	97	**55.4**	102	**58.3**	91	**50.8**
10-14	34	**65.4**	29	**69.1**	31	**70.5**	29	**60.4**	31	**63.3**
**ST9V**										
<1	1	**2.8**	32	**88.9**	23	**74.2**	28	**77.8**	26	**92.9**
1-4	74	**39.6**	108	**65.5**	94	**63.1**	119	**68.4**	89	**54.9**
5-9	147	**76.6**	127	**75.2**	164	**93.2**	138	**77.5**	111	**61.3**
10-14	45	**86.5**	37	**88.1**	44	**97.8**	38	**80.9**	34	**69.4**
**ST14**										
<1	12	**33.3**	34	**94.4**	26	**83.9**	34	**94.4**	23	**82.1**
1-4	84	**46.4**	128	**77.1**	95	**65.1**	114	**65.9**	104	**64.2**
5-9	164	**85.4**	125	**75.8**	131	**76.6**	121	**69.5**	147	**80.8**
10-14	44	**86.3**	40	**95.2**	40	**88.9**	38	**80.9**	38	**82.6**
**ST18C**										
<1	2	**5.6**	32	**88.9**	26	**83.9**	32	**88.9**	22	**78.6**
1-4	52	**27.8**	115	**68.9**	96	**64.9**	85	**48.9**	92	**55.4**
5-9	110	**57.3**	106	**63.1**	152	**86.4**	153	**86.0**	151	**82.5**
10-14	42	**80.8**	36	**85.7**	39	**86.7**	41	**85.4**	46	**93.9**
**ST19F**										
<1	8	**22.2**	33	**91.7**	31	**100.0**	35	**97.2**	27	**96.4**
1-4	118	**65.2**	130	**78.8**	144	**97.3**	158	**91.3**	160	**97.0**
5-9	183	**96.8**	138	**83.1**	172	**99.4**	173	**97.2**	182	**98.9**
10-14	49	**94.2**	41	**97.6**	45	**100.0**	47	**97.9**	49	**100.0**
**ST23F**										
<1	3	**8.3**	21	**60.0**	21	**67.7**	23	**63.9**	16	**57.1**
1-4	30	**16.0**	91	**55.2**	48	**32.0**	69	**39.7**	68	**41.0**
5-9	77	**40.1**	102	**61.5**	118	**67.1**	100	**56.2**	104	**56.5**
10-14	35	**67.3**	31	**73.8**	36	**80.0**	33	**68.8**	27	**55.1**
**Non-vaccine types**										
**6A**										
<1	2	**5.6**	13	**36.1**	8	**25.8**	13	**37.1**	8	**28.6**
1-4	91	**48.9**	124	**75.2**	117	**78.0**	125	**71.8**	137	**82.5**

	5-9	161	**84.3**	160	**94.7**	170	**97.1**	167	**94.4**	177	**96.7**
	10-14	44	**84.6**	42	**100.0**	45	**100.0**	47	**97.9**	48	**98.0**
**19A**											
	<1	9	**25.7**	21	**58.3**	24	**77.4**	27	**75.0**	22	**78.6**
	1-4	119	**65.0**	154	**94.5**	130	**88.4**	161	**94.2**	153	**92.7**
	5-9	176	**92.6**	166	**99.4**	175	**99.4**	177	**100.0**	184	**100.0**
	10-14	49	**94.2**	42	**100.0**	45	**100.0**	48	**100.0**	48	**100.0**

Abbreviations: ST: serotype; VT: vaccine serotypes

Supplementary Figure 1. The proportion of participants with IgG GMCs >0.35 ug/ml, across survey rounds, by age group

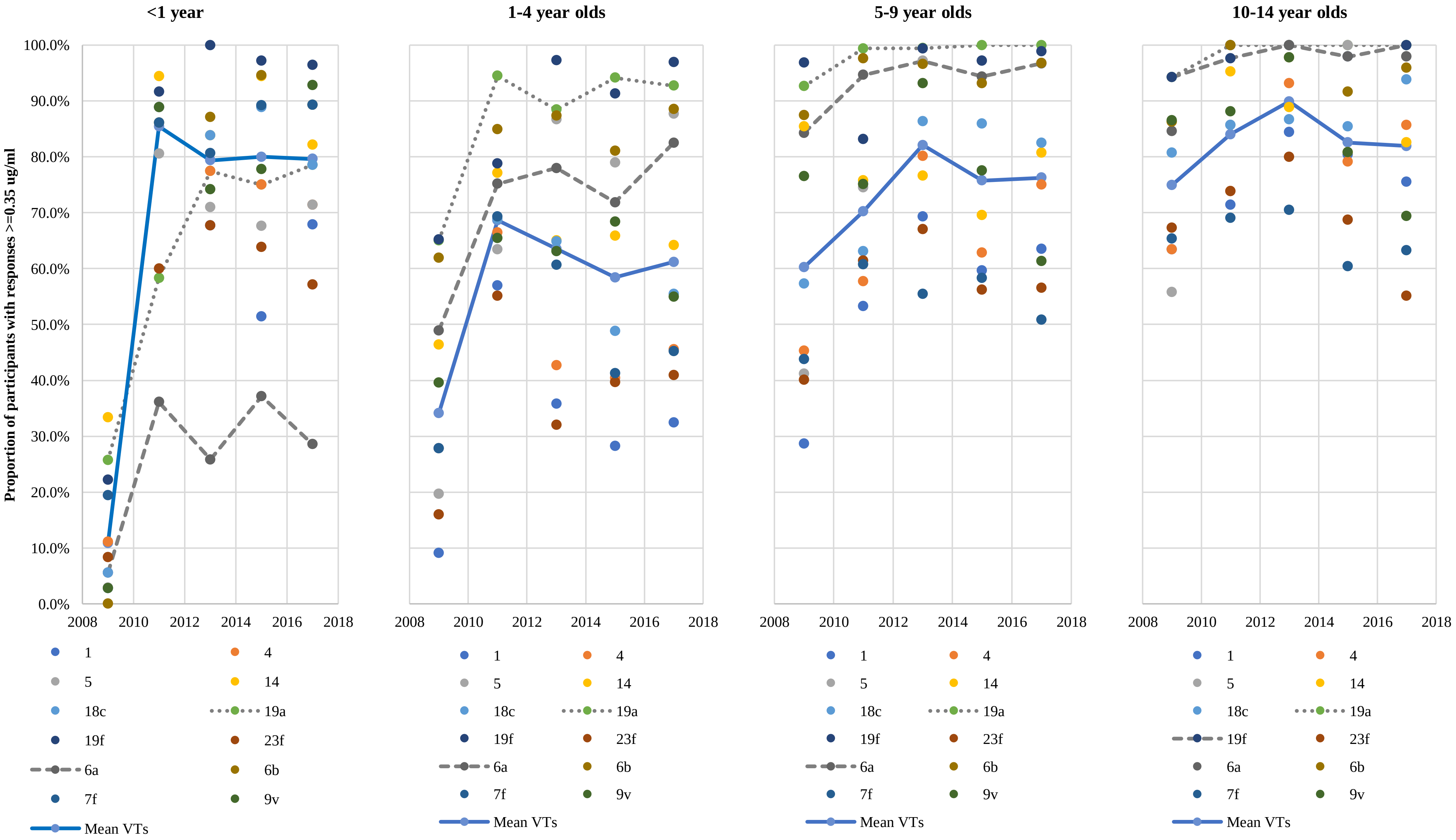

Supplementary Figure 2: Standardised log IgG concentrations (mean z-sores) for all vaccine serotypes combined, by round and age group, with locally weighted regression lines[1]

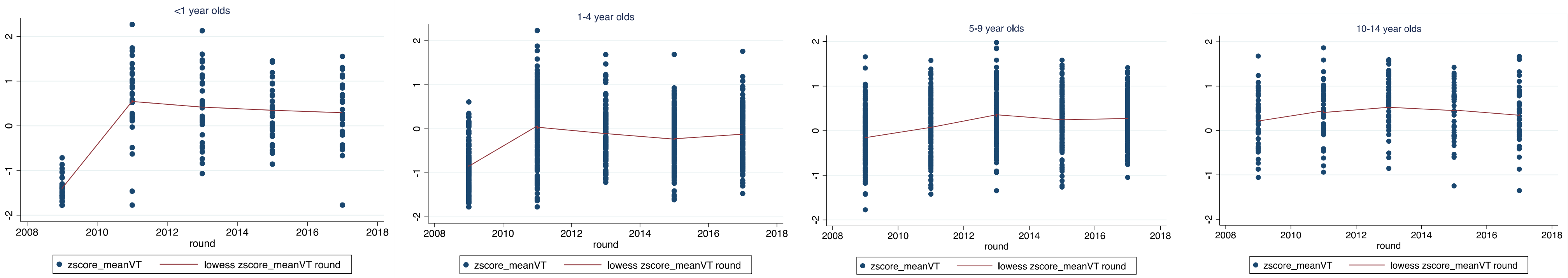

[1] ST specific IgG concentrations were log transformed and converted into a z-score using the formula z= (x-u)/d where x was the raw logGMC, u was the ST-specific mean logGMC (across all time points and age groups) and d was the standard deviation of the stated mean. The 10 z-scores for vaccine serotypes were then combined into a single mean z-score for vaccine types which was plotted by round and age group. A locally weighted linear regression line was overlaid on the plot using the lowess command (STATA Corp. 17). Actual linear regression outputs are provided in supplementary table 2.

Supplementary Table 3: The change in standardised IgG concentrations for all VTs combined by survey round, estimated using linear regression of mean z-scores

Age group	<1 yr olds		1-4 yr olds		5-9 yr olds		10-14 yr olds	
**Survey round**	**Coef**	**95%CI[1]**	**Coef**	**95%CI**	**Coef**	**95%CI**	**Coef**	**95%CI**
**2009**	**-1.98**	**-.2.29, -1.66**	**-0.89**	**-1.02, -0.75**	**-0.23**	**-0.35, -0.11**	-0.26	-0.50, 0.00
**2011**	1		1		1		1	
**2013**	-0.14	-0.47, 0.18	**-0.15**	**-0.29, -0.01**	**0.28**	**0.16, 0.41**	0.19	-0.07, 0.44
**2015**	-0.21	-0.54, 0.10	**-0.28**	**-0.41, -0.14**	**0.17**	**0.04, 0.30**	-0.07	-0.32, 0.19
**2017**	-0.27	-0.60, -0.07	**-0.17**	**-0.30, -0.03**	**0.20**	**0.07, 0.33**	-0.09	-0.34, 0.16

[1]The 95%CI that do not cross the null are highlighted in bold. 2011 was used as the reference year.

Supplementary Table 4. The proportion of children with IgG GMCs >0.35 mg/ml among children vaccinated in infancy (born on or after 1st Feb 2010 and therefore aged<12 months at the time of vaccine introduction on 31st Jan 2011 & who received 3 PCV doses; n=425)

Age (years)	<1		1		2		3		4		5		6	
ST	n >0.35 mg/ml	%	n >0.35 mg/ml	%	n >0.35 mg/ml	%	n >0.35 mg/ml	%	n >0.35 mg/ml	%	n >0.35 mg/ml	%	n >0.35 mg/ml	%
**14**	79	**92.9**	83	**82.2**	37	**50**	42	**67.7**	28	**70.0**	26	**81.2**	14	**73.7**
**1**	63	**75.0**	33	**33.0**	16	**22.9**	17	**29.8**	13	**34.2**	17	**58.6**	8	**44.4**
**18C**	79	**92.9**	60	**60.0**	32	**42.7**	36	**58.1**	21	**51.2**	30	**93.8**	12	**63.2**
**19F**	83	**97.7**	96	**96.0**	67	**90.5**	59	**95.2**	39	**95.1**	31	**96.9**	19	**100**
**23F**	59	**70.2**	33	**32.7**	24	**32.0**	20	**32.3**	24	**58.4**	17	**53.1**	11	**57.9**
**4**	73	**85.9**	40	**39.6**	30	**40.0**	28	**45.2**	18	**43.9**	23	**74.2**	13	**68.4**
**5**	66	**79.5**	69	**69.0**	56	**74.7**	54	**88.5**	38	**95.0**	32	**100**	18	**94.7**
**6B**	81	**94.2**	83	**82.2**	62	**82.7**	55	**88.7**	35	**85.4**	32	**100**	19	**100.0**
**7F**	80	**93.0**	74	**73.3**	33	**44.0**	22	**35.5**	15	**36.6**	18	**58.1**	7	**36.8**
**9V**	72	**84.7**	61	**61.0**	42	**56.0**	36	**58.1**	23	**57.5**	24	**77.4**	11	**61.1**
**6A**	31	**36.9**	57	**57.0**	50	**66.7**	55	**88.7**	38	**92.7**	32	**100**	18	**94.7**
**19A**	65	**76.5**	85	**86.7**	62	**82.7**	60	**96.8**	41	**100.0**	32	**100.0**	19	**100.0**

Supplementary Table 5. Change in ST-specific log (IgG concentrations) and the mean z-score for all VTs, by age, among those vaccinated in infancy

	Mean z-scores for all VTs		ST1		ST4		ST5		ST6B		ST7F		ST9V		ST14	
Age (years)	Coef[1]	95%CI	Coef	95%CI	Coef	95%CI	Coef	95%CI	Coef	95%CI	Coef	95%CI	Coef	95%CI	Coef	95%CI
<1	1															
1	**-.64**	**-0.81, -0.47**	**-0.99**	**-1.27, -0.73**	**-1.06**	**-1.37, -0.76**	**-0.25**	**-0.48, -0.01**	**-0.54**	**-0.82, -0.25**	**-0.82**	**-1.07, -0.57**	**-0.57**	**-0.83, -0.31**	**-0.77**	**-1.15, -0.39**
2	**-.91**	**-1.09, -0.72**	**-1.25**	**-1.54, -0.95**	**-1.13**	**-1.47, -0.80**	0.01	-0.24, 0.26	**-0.66**	**-0.96, -0.36**	**-1.64**	**-1.91, -1.37**	**-0.89**	**-1.17, -0.61**	**-1.53**	**-1.94, -1.12**
3	**-.70**	**-0.90, -0.51**	**-0.95**	**-1.26, -0.63**	**-0.98**	**-1.33, -0.63**	0.40	0.14, 0.67	**-0.36**	**-0.68, -0.04**	**-1.77**	**-2.05, -1.48**	**-0.79**	**-1.08, -0.49**	**-1.08**	**-1.51, -0.64**
4	**-.57**	**-0.80, -0.35**	**-0.62**	**-0.98, -0.26**	**-1.05**	**-1.45, -0.65**	0.77	0.46, 1.08	-0.11	-0.48, 0.25	**-1.70**	**-2.02, -1.38**	**-0.60**	**-0.94, -0.26**	**-0.78**	**-1.27, -0.28**
5	**-.12**	**-0.37, -0.14**	**-0.47**	**-0.86, -0.07**	-0.38	-0.82, 0.05	0.91	0.58, 1.25	0.28	-0.12, 0.67	**-1.31**	**-1.66, -0.96**	0.01	-0.37, 0.38	-0.06	-0.59, 0.47
6	**-.44**	**-0.74, -0.14**	**-0.82**	**-1.29, -0.34**	-0.24	-0.77, 0.29	0.75	0.34, 1.15	0.18	-0.31, 0.66	**-1.71**	**-2.14, -1.28**	-0.68	-1.14, -0.22	-0.47	-1.13, 0.18
7	-.18	-0.65, 0.30	-0.16	-0.88, 0.55	0.07	-0.70, 0.84	0.57	-0.02, 1.16	-0.20	-0.91, 0.51	**-1.83**	**-2.50, -1.17**	-0.36	-1.01, 0.30	-1.02	-1.98, -0.07

	ST18C		19f		23f		6A		ST19A	
Age (years)	Coef	95%CI	Coef	95%CI	Coef	95%CI	Coef	95%CI	Coef	95%CI
<1	1		1		**1**					
1	**-1.28**	**-1.58, -0.98**	**-0.58**	**-0.90, -0.26**	**-0.83**	**-1.19, -0.48**	0.47	0.19, 0.75	0.48	0.16, 0.79
2	**-1.80**	**-2.12, -1.48**	**-0.94**	**-1.28, -0.60**	**-0.77**	**-1.16, -0.40**	0.75	0.45, 1.05	0.68	0.34, 1.01
3	**-1.51**	**-1.85, -1.17**	**-0.37**	**-0.73, -0.02**	**-0.94**	**-1.35, -0.54**	1.36	1.05, 1.68	1.36	1.00, 1.71
4	**-1.70**	**-2.08, -1.31**	**-0.48**	**-0.89, -0.08**	-0.36	-0.81, 0.10	1.51	1.15, 1.87	1.47	1.07, 1.87
5	**-0.95**	**-1.37, -0.53**	0.20	-0.24, 0.64	-0.20	-0.69, 0.30	2.07	1.69, 2.47	2.19	1.75, 2.63
6	**-1.27**	**-1.78, -0.74**	-0.32	-0.87, 0.22	0.03	-0.58, 0.63	1.78	1.30, 2.26	1.87	1.34, 2.41
7	-0.75	-1.51, 0.00	-0.37	-1.16, 0.42	0.08	-0.81, 0.97	1.62	0.92, 2.32	1.79	1.00, 2.57

[1]Coefficients represent the change in the log(IgG) or the mean z-score for all VTs for each year of age, using linear regression, restricted to those vaccinated with 3 doses in infancy. The log IgG concentrations are displayed in Figure 2 in the main text.

Supplementary Figure 3. Standardised IgG concentrations (z-scores) for all VTs among 5-9 yr olds who a) eligible for infant vaccination and received 3 doses in infancy b) eligible for catch up and received 1 or 2 doses in catch up or c) were ineligible for vaccination and received no vaccination

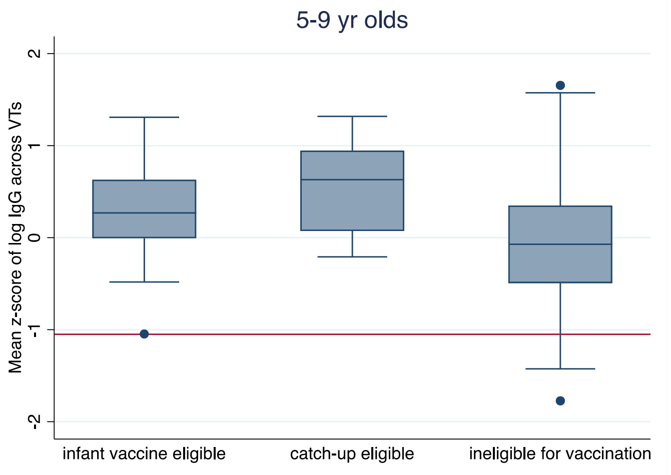

Abbreviations: ST: serotype; VT: vaccine serotypes

Supplementary Table 6: The association between standardised IgG concentrations (z-scores) for all VTs and cohort of vaccination, among 5-9 year olds

Cohort	N (5-9 yr olds)	Mean z-score for all VTs	Coef[1]	95%CI	Alt baseline Coef[1]	95%CI
Received 3 doses of infant vaccine	49	0.31	1		0.50	0.32, 0.68
Received 1 or 2 doses in catch-up	23	0.56	0.21	-0.07, 0.50	0.71	0.46, 0.95
Ineligible for vaccination	300	-0.05	**-0.50**	**-0.32, -0.68**	1	

[1] Coefficients are a result of linear regression of the z-score over cohort, controlling for age

Supplementary Figure 4. Log IgG concentrations among all participants age 10-14 over time[1]. [1]Data for this is in table 2 in the main manuscript

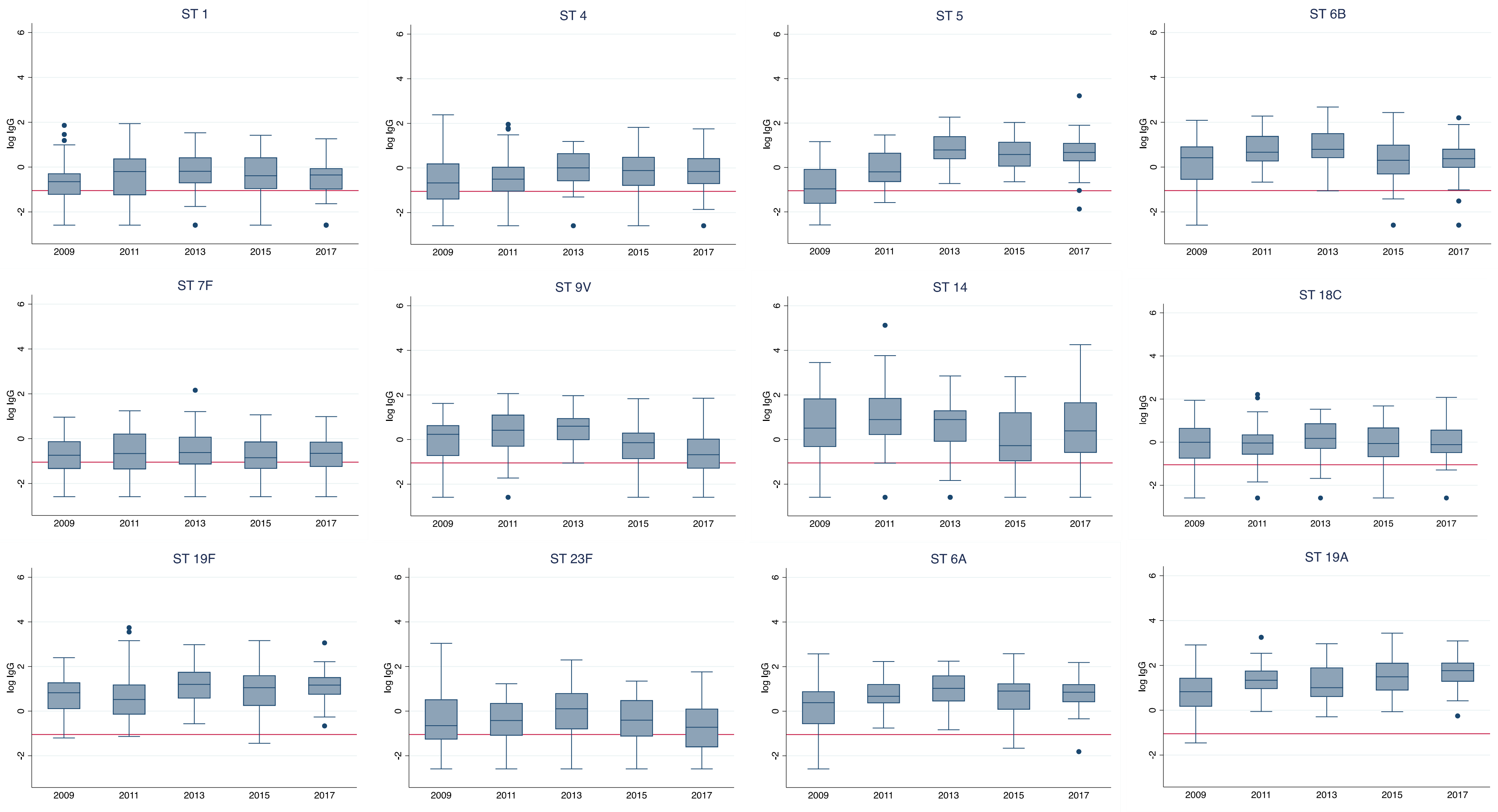

Supplementary Table 7. ST-specific IgG GMCs among participants aged 10-14 who remained unvaccinated, over time

	2009 n=51			2011 n=42			2013 n=45			2015 n=39			2017 n=6			
	GMC	95%CI lb	95%CI ub	GMC	95%CI lb	95%CI ub	GMC	95%CI lb	95%CI ub	GMC	95%CI lb	95%CI ub	GMC	95%CI lb	95%CI ub	p-value[1]
ST14	1.78	1.13	2.79	2.78	1.77	4.38	1.86	1.25	2.76	1.09	0.71	1.67	1.36	0.36	5.16	0.062
ST1	0.50	0.38	0.67	0.72	0.49	1.06	0.82	0.61	1.10	0.84	0.62	1.12	0.67	0.40	1.13	0.100
ST18C	0.94	0.69	1.28	0.89	0.65	1.23	1.10	0.84	1.45	1.08	0.81	1.43	0.81	0.43	1.51	0.790
ST 19F	2.00	155	2.58	2.05	1.41	2.99	3.15	2.41	4.12	3.10	2.26	4.26	3.08	1.66	5.73	0.064
ST 23F	0.64	0.44	0.94	0.72	0.53	0.96	1.00	0.71	1.40	0.73	0.52	1.04	0.39	0.21	0.73	0.210
ST 4	0.53	0.37	0.75	0.69	0.50	0.97	0.99	0.77	1.26	0.85	0.60	1.20	0.87	0.44	1.70	0.055
ST 5	0.38	0.29	0.52	0.95	0.74	1.23	2.30	1.84	2.87	1.97	1.56	2.47	1.87	0.87	4.04	<0.001
ST6B	1.23	0.92	1.65	2.14	1.69	2.71	2.29	1.79	2.93	1.44	1.05	1.97	1.27	0.47	3.43	0.004
ST7F	0.43	0.32	0.56	0.52	0.37	0.72	0.56	0.40	0.76	0.49	0.36	0.66	0.51	0.33	0.79	0.760
ST9V	1.06	0.83	1.37	1.34	0.97	1.86	1.60	1.28	2.01	0.81	0.63	1.04	0.30	0.12	0.74	<0.001
Non-vaccine STs																
ST 6A	1.12	0.85	1.49	1.97	1.59	2.45	2.61	2.07	3.29	2.29	1.76	2.98	2.34	0.90	6.09	<0.001
ST19A	2.25	1.68	3.01	4.00	3.26	4.91	3.46	2.71	4.42	4.95	3.88	6.31	6.17	3.57	10.6	0.001

Abbreviations: CI: confidence interval; GMC: geometric mean concentration; lb: lower bound; ub: upper bound; ST: serotype; VT: vaccine serotypes
[1]p-values are derived from linear regression likelihood ratio tests of changes in log(IgG) concentrations by survey round, in each age group.

Supplementary Figure 5. Standardised mean z-scores for all VTs among: left) all participants aged 10-14, and; right) those who remained unvaccinated, over time

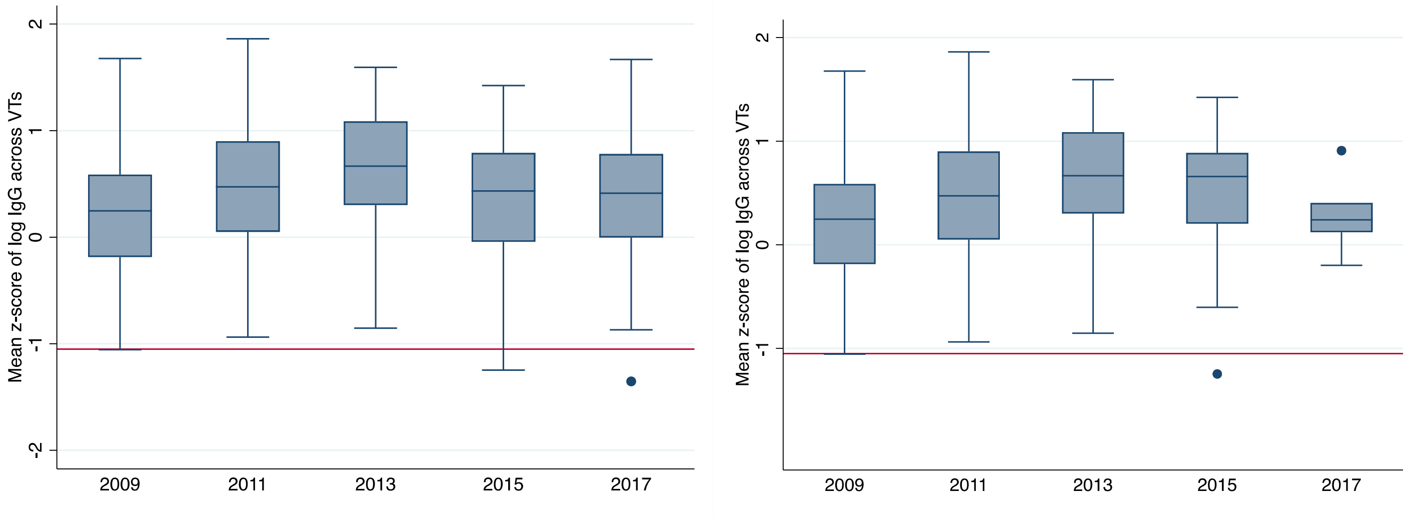

Abbreviations:ST: serotype; VT: vaccine serotypes

Supplementary Table 8. Linear regression coefficients. Mean standardised z-score of VT GMCs among children 10-14 years of age who were ineligible for vaccination and unvaccinated, by round

Round	N	Mean z-score	Linear reg Coef[1]	95% CI
2009	50	0.21	**-0.24**	**-0.48, 0.01**
2011	42	0.46	1	
2013	43	0.65	0.21	0.04, 0.46
2015	37	0.51	0.01	-0.26, 0.27
2017	5	0.29	-0.27	-0.83, 0.29

Abbreviations: CI: confidence interval; GMC: geometric mean concentration; lb: lower bound; ub: upper bound; ST: serotype; VT: vaccine serotypes.
[1] controlling for age

Supplementary Figure 6: Graphical representation of the number sampled per survey round and contributions to the birth cohort analysis.

**Survey year:**	**2009**	**2011**	**2013**	**2015**	**2017**	
**Age at sampling**						**Total**
**10-14 years**	52	42	45	48	49	236
**8-9 years**	49	51	51	49	43	243
**7 years**	48	36	35	48	44	211
**6 years**	47	44	48	34	48	221
**5 years**	48	39	43	47	49	226
**4 years**	53	47	47	44	36	227
**3years**	44	40	33	46	50	213
**2 years**	50	39	36	40	39	204
**1 year**	42	41	34	44	42	203
**<1 year**	36	36	31	37	28	168
**Total**	**469**	**415**	**403**	**437**	**428**	**2,152**

Key:

(white)	Ineligible for vaccination
(green)	Eligible for vaccination in infancy
(orange)	Eligible for vaccination in catch-up (approximate)
